# Supplementary material for: Time-Series Metabolome and Transcriptome Analyses Reveal the Genetic Basis of Vanillin Biosynthesis in Vanilla
Source: Plants (Basel). 2025 Jun 23;14(13):1922. doi: 10.3390/plants14131922 (PMC12252122; doi:10.3390/plants14131922)
Supplement: Supplementary file 1 [file plants-14-01922-s001.zip › Supplementary Figure.pdf]

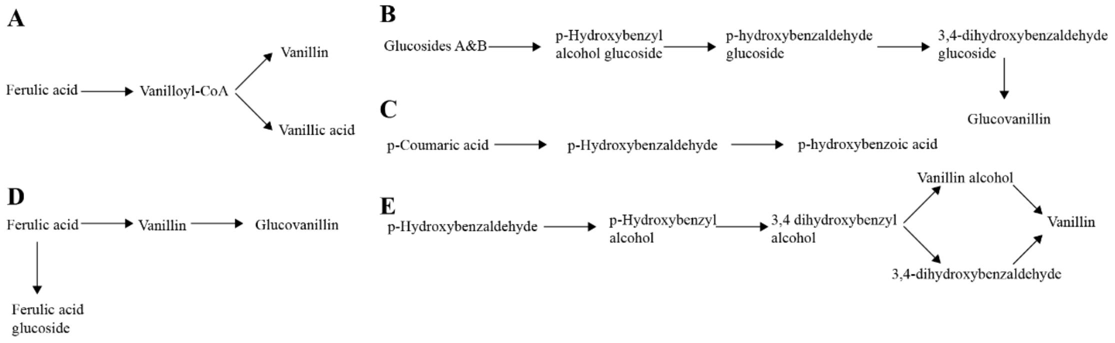

Figure S1 Multiple routes have been proposed to describe the vanillin biosynthetic pathway. (A) Zenk vanillin pathway in plants (Kundu, 2017). Oxidative conversion of ferulic-CoA to vanilloyl-CoA followed by reduction to vanillin. Catalytic enzymes remain unidentified. (B) Kanizawa vanillin pathway in plants (Kanisawa, Tokoro and Kawahara, 1994). UGT gene family plays key role in the synthesis pathway. (C) Yazaki non oxidative chain shortening pathway in plants (Yang et al., 2017). p-Coumarate → 4-hydroxybenzaldehyde (4HBA) conversion catalyzed by thiol-dependent 4HBS (non-oxidative chain shortening). *V. planifolia* pods accumulate 4HBA-glucoside, requiring P450-mediated 3-hydroxylation and O-methylation for vanillin biosynthesis. (D) Benzoate route for vanillin synthesis in plants (Havkin-Frenkel and Belanger, 2011). Oxidation was considered as the mechanism for the conversion of ferulic acid to the vanillin and glucovanillin. (E) Ferulic acid → vanillin *VpVAN* mediated pathway in plants (Gallage et al., 2014). *VpVAN* was considered playing key role for the conversion of ferulic acid to the vanillin.

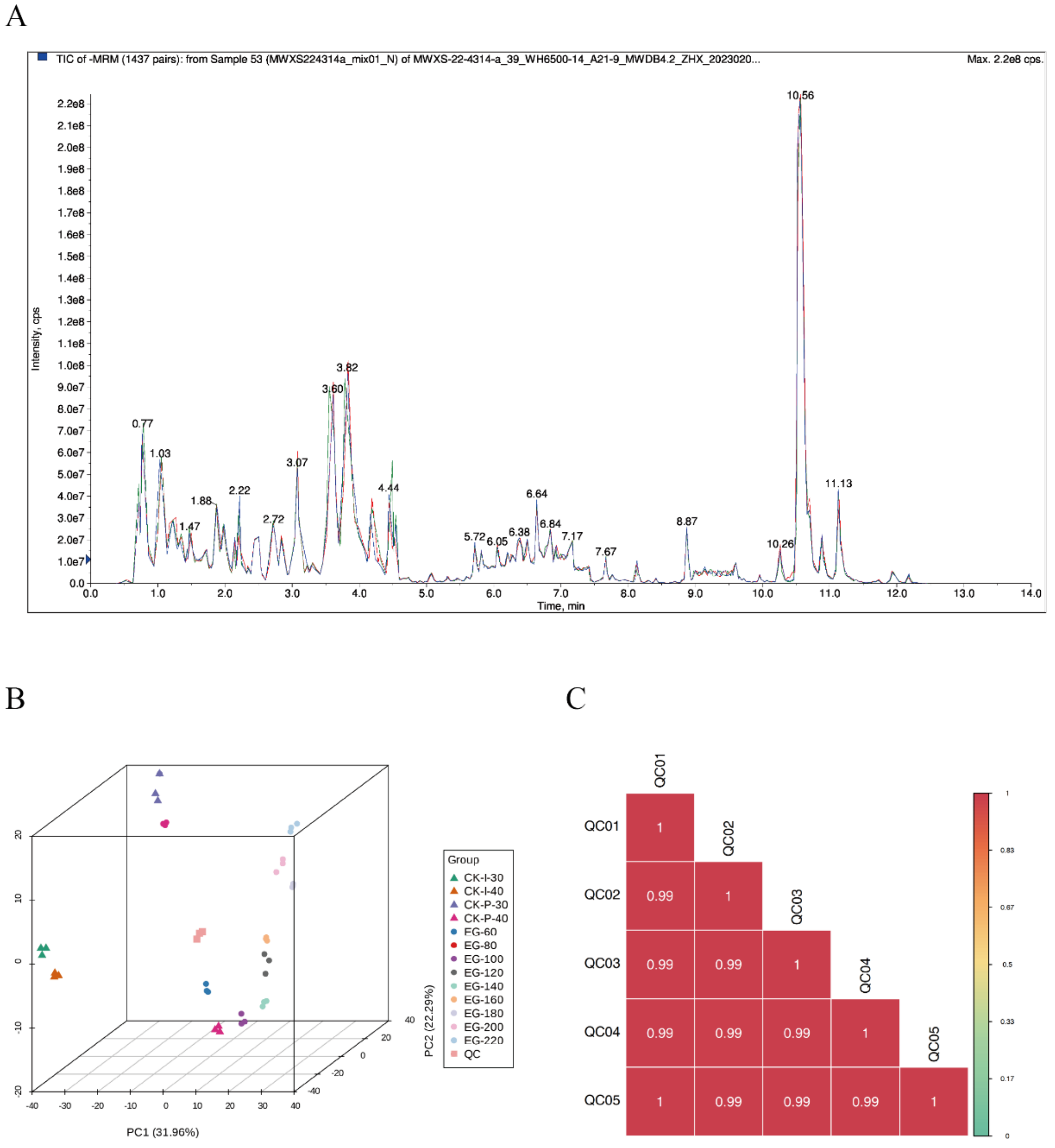

Figure S2. Quality assessment of metabolomics data. (A) Overlay of total ion chromatograms (TICs) from quality control (QC) samples in mass spectrometry analysis, showing high reproducibility across repeated measurements. (B) Percentage values indicate the proportion of variance explained by each principal component in the dataset. CK denotes control species with low vanillin content *Vanilla imperialis* group, while EG indicates high-vanillin *Vanilla planifolia* group. The significant separation between CK and EG along PC1 (explaining 31.96% variance) confirms metabolic profile differences, validating the detection precision. (C) Color gradient represents Pearson correlation coefficients, with deeper red hues indicating stronger positive correlations among samples.

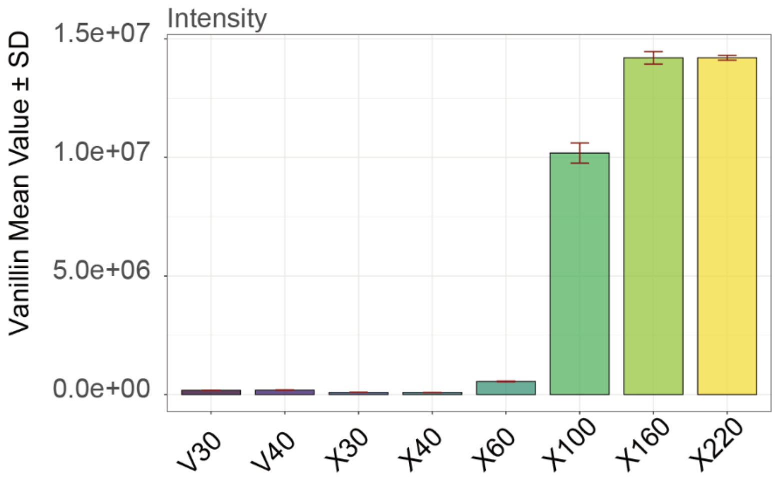

Figure S3. Fluctuations in vanillin accumulation patterns across developmental stages.

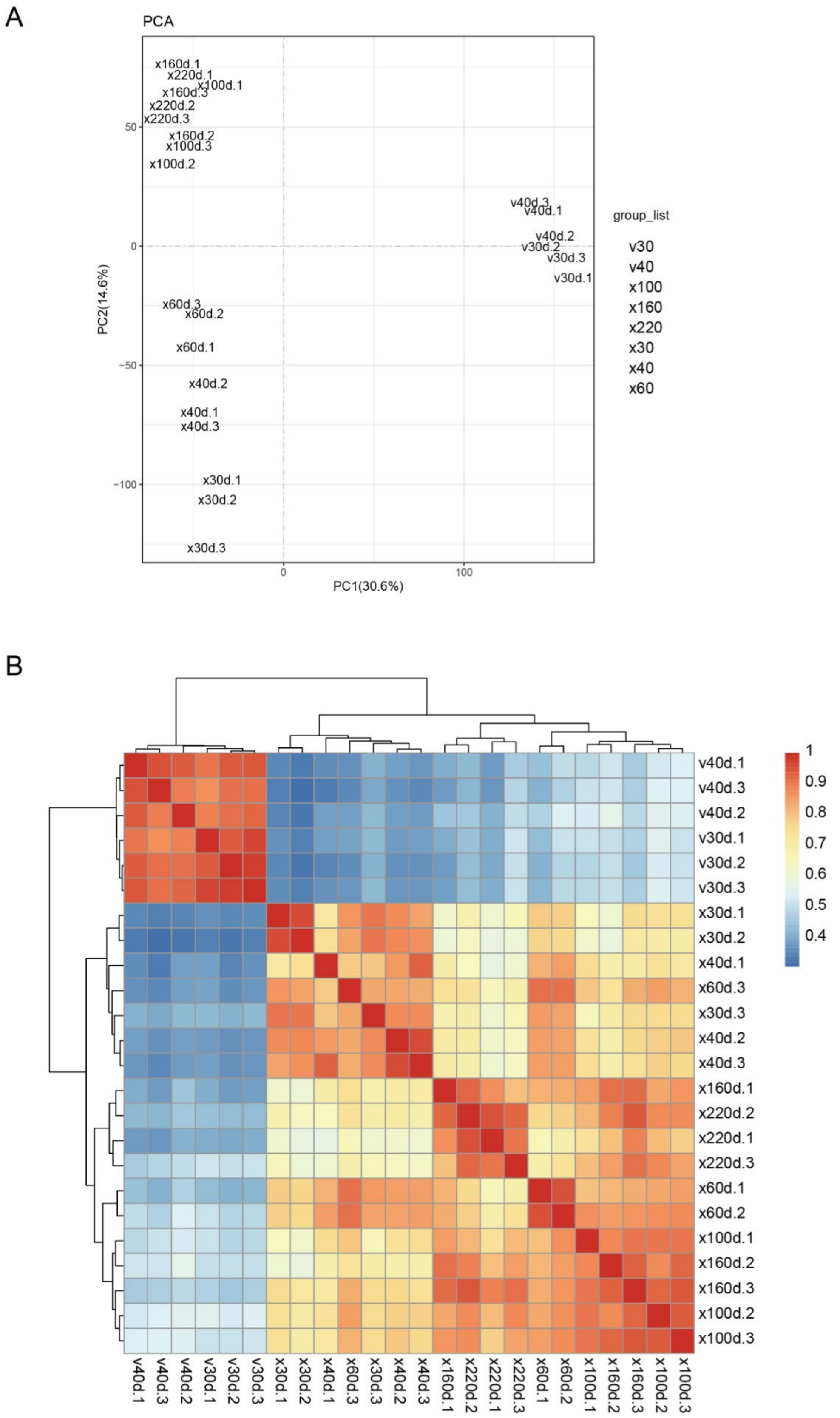

Figure S4. Quality assessment of transcriptome data. (A) Principal component analysis (PCA) of samples showing their clustering patterns. (B) Heatmap of correlation coefficients among samples demonstrating pairwise transcriptomic relationships.

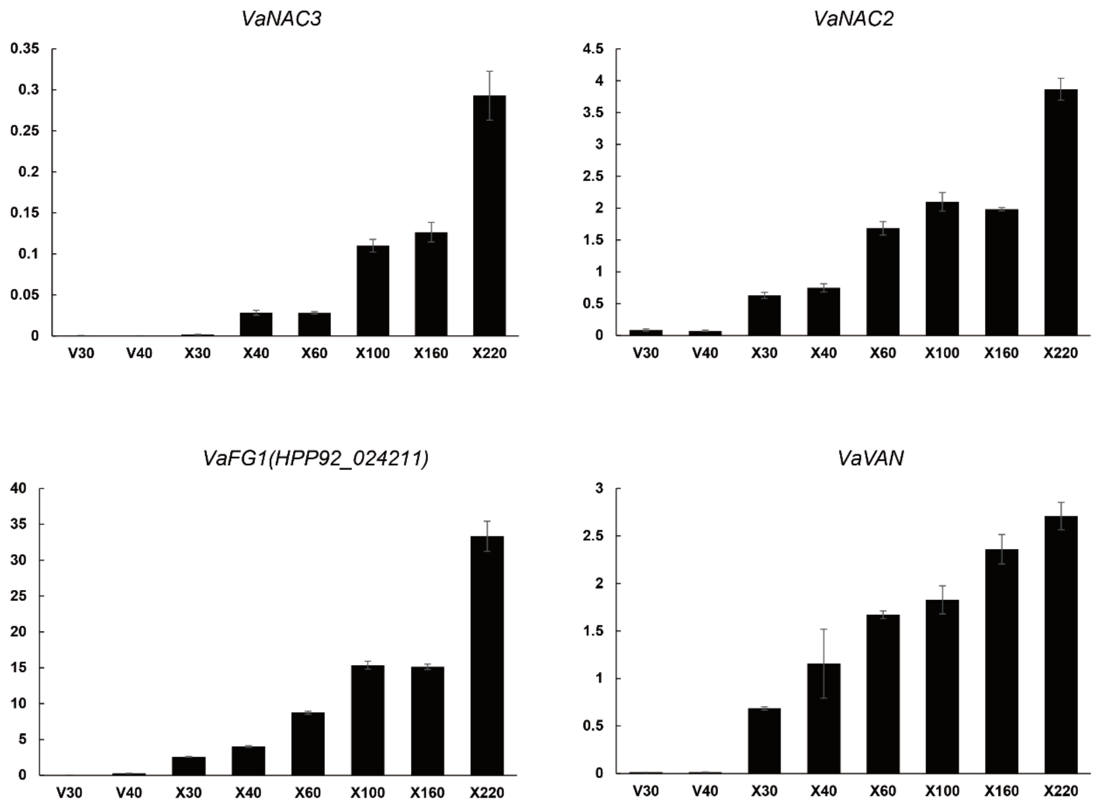

Figure S5. qPCR validate for some DEGs.

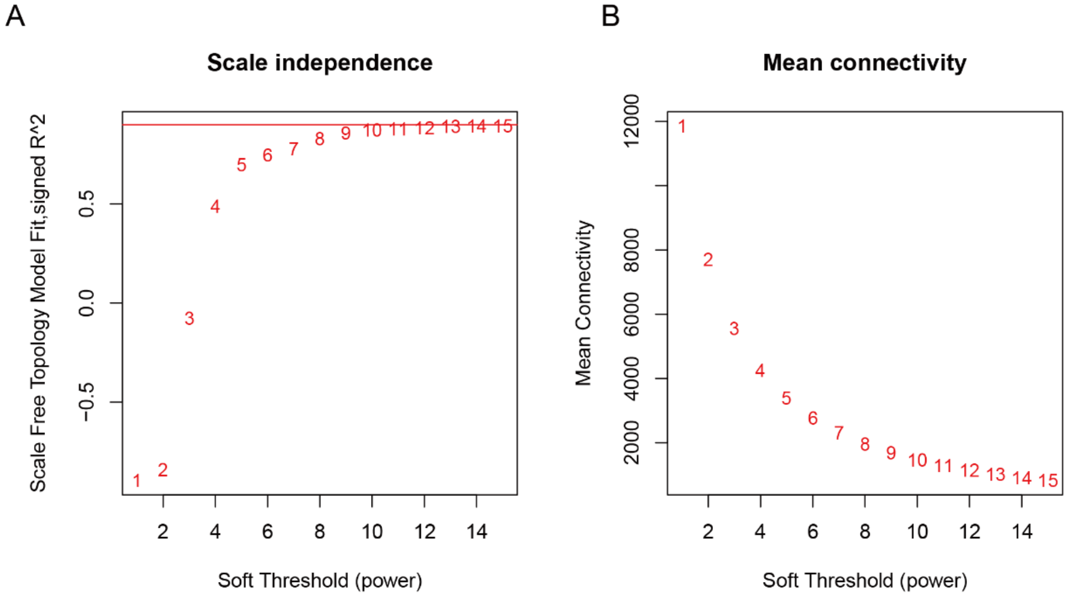

Figure S6. Determination of soft-thresholding power and module clustering. (A) Scale-free topology fitting index curve; (B) Mean connectivity curve under scale-free criteria.
